# A Wearable Integrated Microneedle Electrode Patch for Exercise Management in Diabetes

**DOI:** 10.34133/research.0508

**Published:** 2024-10-21

**Authors:** Boyu Zhu, Lihang Zhu, Xinru Li, Ziyi Zhao, Jiayi Cao, Min Qi, Zhigang Gao, Lin Zhou, Bin Su

**Affiliations:** ^1^ Institute of Analytical Chemistry, Department of Chemistry, Zhejiang University, Hangzhou 310058, China.; ^2^ Department of Clinical Engineering, Second Affiliated Hospital, College of Medicine, Zhejiang University, Hangzhou 310009, China.; ^3^ General Surgery Department, Children’s Hospital, Zhejiang University School of Medicine, National Clinical Research Center for Child Health, Hangzhou 310052, China.

## Abstract

Exercise is one of the preferred management strategies for diabetic patients, but the exercise mode including type, intensity, and duration time is quite different for each patient because of individual differences. Inadequate exercise has no effect on the blood glucose control, while overexercise may cause serious side effects, such as hypoglycemia and loss of blood glucose control. In this work, we report a closed-loop feedback mode for exercise management in diabetes. A minimally invasive, biocompatible microneedle electrode patch was fabricated and used for continuously monitoring the glucose in the interstitial fluid. Further, in conjunction with using a wireless electrochemical device, the glucose signals can be analyzed to output the potency of exercise and give advice on exercise management. A custom exercise given by this closed-loop feedback mode can reduce the used dose of insulin and avoid side effect during and after exercise. We believe that this work can provide a novel comprehensive guidance for diabetic patients.

## Introduction

Diabetes is one of the most common metabolic disorders, which is mainly caused by insulin hyposecretion or insulin resistance and characterized by high blood glucose level [1]. Diabetes affects hundreds of millions of people worldwide due to its severe complications. Effective diabetes management including blood glucose level monitoring and diabetes treatment is so far the main way of prolonging the lifetime and improving the life quality of diabetic patients [2]. Traditional fingertip blood glucose monitoring is invasive and may easily overlook hyperglycemia/hypoglycemia [3]. Interstitial fluid (ISF) is formed by the filtration of blood through blood capillaries, and the glucose concentration in ISF is highly correlated with that in blood [4,5]. Therefore, numerous continuous glucose monitoring (CGM), such as Freestyle Libre of Abbott, G6 of Dexcom, and MiniMed 670G of Medtronic, have been developed for continuously monitoring the glucose concentration in ISF to indirectly reflect that in blood [6]. However, the implantation of CGM sensors often requires the assistance of a long needle (~5 mm in length, ~0.5 mm in diameter) to puncture the skin, which may cause pain and tissue infection [7].

Microneedle electrode patch (MEP) is a sort of microelectrode array made up of miniaturized needle-like microelectrodes with a couple of hundred micrometers in length [8–11]. Because of its minimally invasive nature, MEP has been used to pierce skin and electrochemically detect different substances in ISF [12–14]. For example, Wang and colleagues have fabricated a series of wearable MEP for real-time monitoring of glucose, lactate, and drugs during diet, physical exercise, and disease treatment [15,16]. Cui and colleagues have combined MEP with a micropump for insulin administration to construct a closed-loop system for diabetes management [17,18]. Voelcker and colleagues have developed a MEP based on high-density silicon microneedles for painless glucose monitoring in ISF [19]. The transdermal MEP with a minimal invasion holds great promise in painless and continuous glucose monitoring. Although a lot of researches on electrochemical microneedle glucose monitoring systems have been reported, they are rarely applied in the real-world scenarios.

Exercise is a kind of effective adjuvant treatment for diabetes [20–22]. It is usually used together with insulin, which is the most commonly used drug for diabetes treatment [23–26]. Exercise can not only enhance the ingestion and consumption of glucose but also increase the insulin sensitivity [27]. As previously reported, the blood glucose level of type 1 diabetic patients can be decreased by 1- to 2-fold after 1 h of aerobic exercise [28]. However, inadequate exercise has no effect on the blood glucose control and overexercise possibly leads to a variety of side effects, such as hypoglycemia and even patient death [29]. Therefore, a management system or mode capable of advising exercise type, intensity, or duration time is urgently required [30,31].

**Fig. 1.**
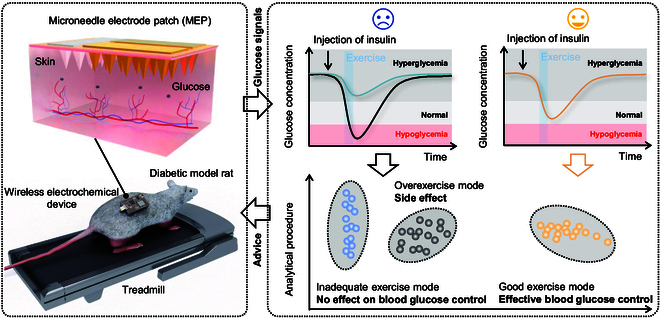
Schematic illustration of the closed-loop feedback system for exercise management in diabetes.

Herein, we report a closed-loop feedback system for exercise management in diabetes. As illustrated in Fig. 1 (left panel), a microdevice consisting of a MEP and a battery-powered custom potentiostat was fabricated and fixed on the freely moving rat for continuous and real-time monitoring of the glucose in ISF during running. The glucose signals wirelessly collected by a smart mobile phone could be analyzed by a user interface to classify the potency of exercise mode and insulin dose (right panel in Fig. 1). The objective is to optimize and advise a good exercise mode for individual patients to realize personalized and custom management in diabetes, which is expected to reduce the drug dose and avoid unwanted side effects (such as hypoglycemia due to overexercise).

## Results

### MEP for glucose detection

**Fig. 2.**
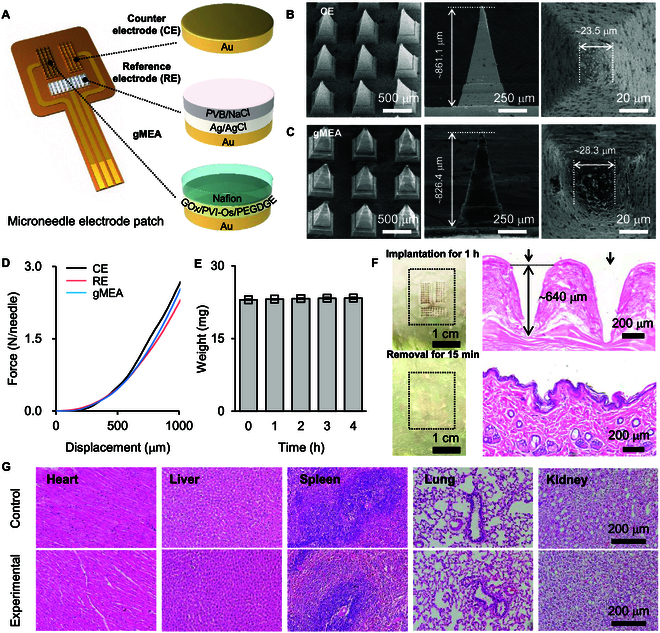
Characterization and biocompatibility of the MEP. (A) Schematic structure of the MEP for glucose sensing (left) and the composition of each electrode (right). (B) Top-view scanning electron microscopy (SEM) images of MEA (namely, CE) (left), side-view SEM images (middle), and high-magnification SEM images of the tip (right) of a single microneedle. (C) Top-view SEM images of gMEA (left), side-view SEM images (middle), and high-magnification SEM images of the tip (right) of a single microneedle. (D) Force–displacement curves obtained with CE (black), RE (red), and gMEA (blue). (E) Weight of MEP immersed in 1× PBS for different times (*n* = 3 MEPs). (F) Photographs of rat skin (left) and H&E-stained rat skin sections (right) after implanting MEP for 1 h (top) and after removal of MEP for 15 min (bottom). The dotted boxes and arrows annotate the implanted locations of microneedles. (G) Optical images of H&E-stained heart, liver, spleen, lung, and kidney sections from normal rats (control group) and rats implanted with MEP for 5 h (experimental group).

Figure 2A shows the structure of MEP sensor for electrochemical monitoring of glucose in ISF. The hydrophobic polystyrene (PS) was used first to prepare the underlying microneedle array, on which a thin gold layer was then electrolessly deposited to prepare the microneedle electrode array (MEA). A MEA, a silver/silver chloride (Ag/AgCl) ink-coated MEA, and a glucose sensing layer coated MEA (gMEA) functioning as counter, reference, and working electrodes (CE, RE, and WE, respectively) were assembled on a polyimide (PI) substrate to obtain the MEP sensor (see the fabrication details in Figs. S1 to S3). As displayed in Fig. 2B, the height and the tip width of microneedles in MEA (namely, CE) are ~823.3 ± 33.0 and ~26.1 ± 1.8 μm, respectively. The electrolessly deposited gold layer covers evenly the surface of PS microneedle array and shows a high electrochemical activity (Figs. S4 and S5). The glucose sensing layer of gMEA is composed of osmium-derivatized poly(1-vlnylimidazole) (PVI-Os) as the electron mediator, glucose oxidase (GOx) as the biocatalytic element, poly(ethylene glycol) diglycidyl ether (PEGDGE) as the cross-linking agent, and Nafion as the antibiofouling layer [32]. As illustrated in Fig. 2C, the structure of gMEA is similar to that of bare MEA. Both gold and osmium elements uniformly distribute on gMEA (Fig. S6). Finally, a MEA coated with Ag/AgCl ink and polyvinyl butyral (PVB) functions as the solid-state RE, which has a stable potential response (Figs. S7 and S8). Mechanical strength, swelling behavior, and biocompatibility of the microneedles were then evaluated. Figure 2D displays the force–displacement curves recorded with a texture analyzer. All microneedles exhibit an outstanding mechanical strength with a mean compression force of ~0.45 N/needle at a displacement of 500 μm, which is sufficient for the microneedles to penetrate the skin [33]. The swelling nature of microneedles was examined by measuring the variations of their weight after immersion in 1× phosphate-buffered saline (PBS) solution for 4 h (Fig. 2E and Fig. S9). The swelling ratio of whole MEP was only ~2%, indicating a poor swelling capacity and thus a good structural stability. To probe the biocompatibility, the MEP was implanted into the rat skin for 1 h and then skin tissue sections were isolated for hematoxylin and eosin (H&E) staining. The H&E staining images show that the penetration depth of microneedles was ~640 μm (Fig. 2F), which is larger than the thickness of rat epidermis (~20 to 60 μm) but smaller than that of epidermis and dermis (~700 to 800 μm), suggesting that the microneedles were implanted into the dermis layer [34–36]. After removal of MEP from the skin, the microneedle pinholes almost disappeared within 15 min (Fig. 2F and Fig. S10). The morphology of sections from heart, liver, spleen, lung, and kidney of normal rats and rats implanted with MEP for 5 h or 7 d has no obvious difference, further proving the high biocompatibility of MEP (Fig. 2G and Fig. S11).

**Fig. 3.**
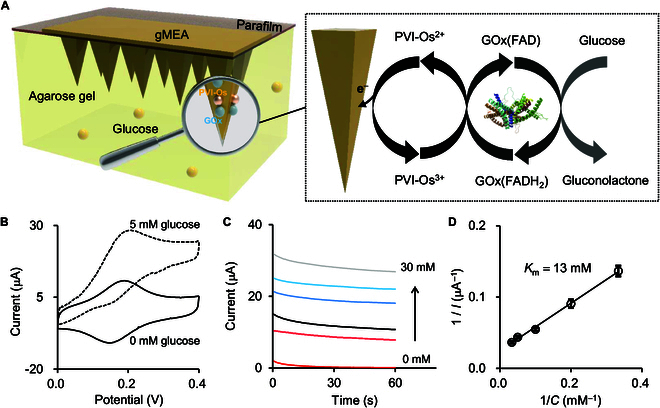
In vitro glucose detection. (A) Schematic illustration of the glucose sensing by MEP in the skin-mimicking agarose gel (left) and the glucose sensing mechanism (right). (B) CVs obtained with MEP in the skin-mimicking agarose gel containing 0 (solid curve) and 5 mM (dashed curve) glucose. The scan rate was 10 mV s^−1^. (C) Chronoamperometric curves recorded with MEP in the skin-mimicking agarose gel containing different concentrations of glucose: 0, 3, 5, 10, 20, and 30 mM from bottom to top. (D) Lineweaver–Burk plot of 1/*I* versus 1/*C* (*n* = 3 times).

Regarding the electrochemical performance of MEP, it was first investigated in skin-mimicking agarose gel [37]. The parafilm and agarose gel imitate the epidermis and dermis of skin (Fig. 3A). Figure 3B shows cyclic voltammograms (CVs) obtained with MEP in the absence and presence of 5 mM glucose. Typical oxidation peak associated with PVI-Os transition is observed at +0.20 V (the same in the PBS solution; Fig. S12A). Glucose reacts with PVI-Os via the catalysis of GOx; therefore, the oxidation current clearly increases with the concentration of glucose (Fig. 3C and Fig. S12B). The apparent Michaelis–Menten constant (*K*_m_) characterizing the enzyme–substrate kinetics can be calculated according to the Lineweaver–Burk equation, which is ~13 mM in the agarose gel (Fig. 3D) and close to that in the PBS solution (~12 mM; Fig. S12C). Considering that numerous substances in ISF might interfere with the glucose detection, the selectivity of MEP was studied in PBS solutions containing interfering substances. As shown in Fig. S13, no obvious current responses (<8.6% with respect to that of 5 mM glucose) were recorded in all cases. The high selectivity of MEP is sufficient to make sure that the current response is dominated by glucose rather than other substances in the ISF. The stability of MEP was further examined by comparing the current response to 5 mM glucose before and after long-term storage and physical bending. A slight decrease in the current response by ~7.2% was recorded after storage at 4 °C under nitrogen (N_2_) atmosphere for 20 d (Fig. S13). An insignificant decrease in the current response by ca. 1.2% to 5 mM glucose were observed when the MEP was physically bended from 0° to 90° (Fig. S14). These results suggest that MEP has excellent electrochemical performance and stability.

### Correlating the current with blood glucose concentration

**Fig. 4.**
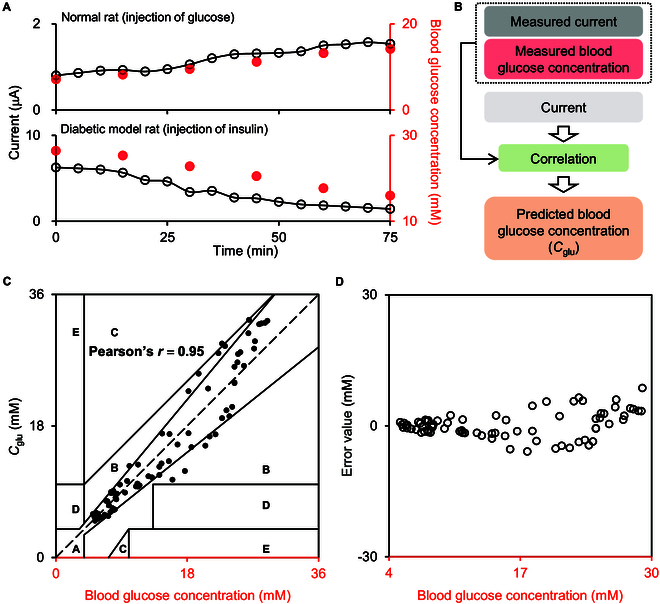
Correlation between the current of MEP and blood glucose concentration. (A) The variation of current recorded with MEP (black) and that of blood glucose concentration measured by a commercial glucometer (red) for normal rat injected with glucose (1 g kg^−1^) and type 2 diabetic model rat injected with insulin (1 U kg^−1^). (B) Workflow used for converting the current response recorded with MEP to *C*_glu_. (C) Clarke error grid for classifying the *C*_glu_ and measured blood glucose concentration. (D) Error values between *C*_glu_ and measured blood glucose concentration.

The correlation between current response and blood glucose concentration can be built for both normal rats and type 2 diabetic model rats. Type 2 diabetic model rats were established by combining high-fat diet and streptozotocin administration (Figs. S15 to S17) [38]. Figure 4A shows the variations of current response in ISF measured by MEP and the blood glucose concentration in the tail vein of rats determined by glucometer. For normal rats injected with glucose (1 g kg^−1^) and diabetic model rats injected with insulin (1 U kg^−1^), current and blood glucose concentration follow the same trend and display a strong correlation (Figs. S18 and S19). The current valued by MEP can be converted to a predicted blood glucose concentration (*C*_glu_, as illustrated in Fig. 4B). Clarke error grid was used for classifying *C*_glu_ and measured blood glucose concentration [39]. As shown in Fig. 4C, 84.21% of data fall in region A, indicating that the difference between 2 values is less than 20%; 14.47% of data is located in region B, meaning that *C*_glu_ can still represent the blood glucose level, although the difference between 2 values is larger than 20%. The rest of 1.32% distributes in other regions as the difference between 2 values is too big. The Pearson’s *r* of 2 datasets was estimated to be ~0.95. Furthermore, the error value between *C*_glu_ and measured blood glucose concentration is smaller than 1.5 and 6.0 mM, respectively, in the concentration ranges of ~4 to 12 mM and ~12 to 30 mM (Fig. 4D). The mean absolute relative difference (MARD) was calculated to be 13.78%. These results demonstrate that MEP current and blood glucose concentration have a strong correlation and *C*_glu_ converted from the current measured by MEP can represent the blood glucose concentration.

### Exercise management in diabetes

**Fig. 5.**
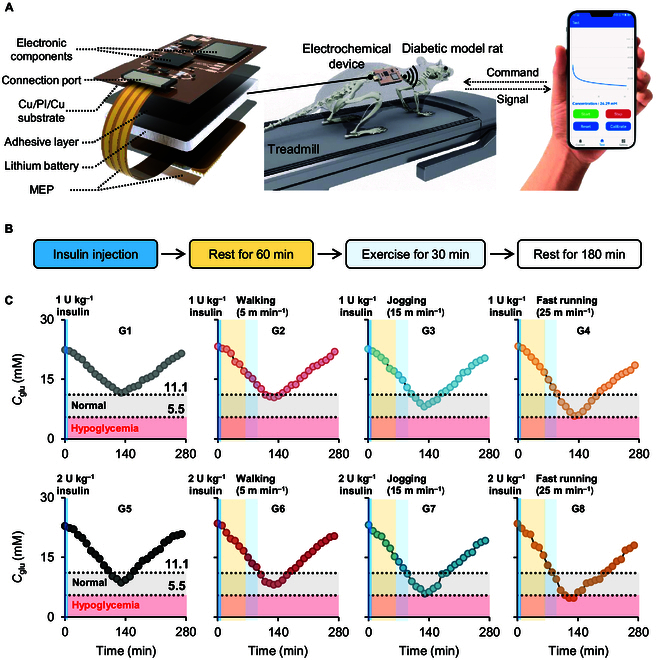
Exercise management using the wireless electrochemical device in diabetic model rats. (A) Exploded view of the custom wireless electrochemical device for glucose detection and schematic illustration of the running exercise management in diabetic model rat. (B) Flowchart of exercise management combined with drug administration. (C) Variation of *C*_glu_ for diabetic model rats in G1 to G8.

Exercise is one of the most effective nonpharmacological adjuvant therapies for diabetes [40]. It can promote the translocation of glucose transporter type 4 (GLUT4, an insulin-responsive transporter protein) from vesicles to cell membrane and increase the insulin sensitivity [41,42]. We intend to develop a closed-loop strategy, in which MEP is integrated with a battery-powered custom potentiostat to continuously monitor the glucose level and the glucose signal is analyzed to provide advice on the exercise management. Figure 5A shows the exploded view of whole device, including MEP, lithium battery, adhesive layer, Cu/PI/Cu substrate, connection port, and electronic components. The size and weight of whole device are ~4.5 × 2.0 × 1.5 cm^3^ and ~7.3 g, respectively, which is light and small enough to be stuck to the rat back and allows the rat to move freely (Movie S1). The power consumption of the device is controlled at 7.0 mW. The lithium battery (150 mA h, 3.7 V) could power this device to work for ~79 h. Moreover, the total cost of the device is $10. The circuit schematic diagram is shown in Figs. S20 and S21. Data transmission is executed via Bluetooth communication, and the current can be converted to *C*_glu_ by an Android app on the smart phone (Fig. S22). The technical performance of the device is comparable to a commercial workstation (Fig. S23). The device was fixed on the back of rat, because this location is relatively flat and the hard skeleton can keep the stability of the device during exercise. Moreover, the chronoamperometric curve was filtered and smoothed to remove the background noise (Fig. S24). The *C*_glu_ measured by MEP on rats running at different speeds is well matched with the blood glucose concentration determined by the commercial FreeStyle Libre glucose sensor (Fig. S25). It can work stably and continuously for 4 h on both freely moving normal and diabetic model rats (Fig. S26). These results suggest that the current can be real-time measured and converted to predicted blood glucose concentration with this wireless electrochemical device.

Figure 5B shows the flow diagram of exercise management combined with drug administration in type 2 diabetic model rat. Briefly, insulin (1 or 2 U kg^−1^) was injected into the abdominal cavity of rat [43–46]. After 60 min, rat rested or exercised on an animal treadmill at a speed of 5, 15, or 25 m min^−1^ for 30 min (designated as resting, walking, jogging, and fast running) (Movie S2). Finally, rat was immediately moved back to its home cage for 180 min. The groups of injection of 1 U kg^−1^ plus resting, walking, jogging, and fast running for 30 min were designated as G1 to G4. Similarly, the groups of injection of 2 U kg^−1^ plus resting, walking, jogging, and fast running for 30 min were designated as G5 to G8. As shown in Fig. 5C, the injection of insulin led to a gradual decrease of *C*_glu_ lasting for ~130 min and then an inverse increase of *C*_glu_. In G5, *C*_glu_ can decline to the normal concentration range (~5.5 to 11.1 mM, the gray region) [47]. As for the experimental group, the hypoglycemic efficacy of different exercise modes is quite different. Walking seems to have no obvious effect on the blood glucose control, and the variation of *C*_glu_ is very similar to that of the control group. Upon increasing the exercise intensity (running speed), *C*_glu_ declines more significantly. Moreover, the concentration range (gray region) apparently increases as well. However, it is also found that fast running will cause the risk of hypoglycemia (<5.5 mM, pink region).

**Fig. 6.**
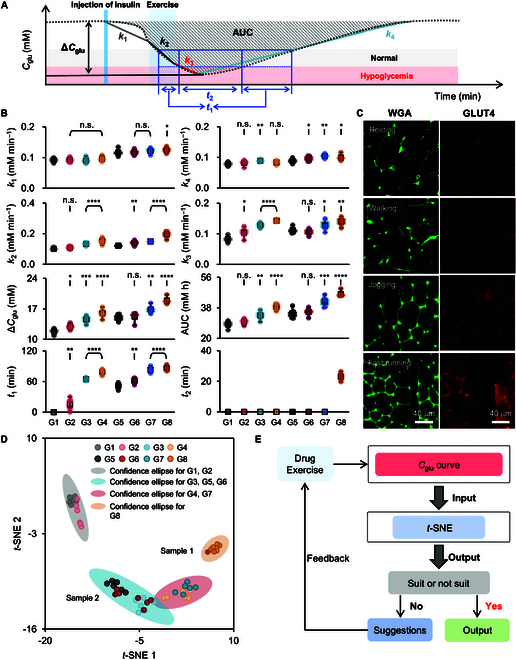
Statistical analysis of dynamic variation of *C*_glu_ and the closed-loop feedback mode for diabetes management. (A) Analytical model for evaluating the dynamic variation of *C*_glu_ in terms of 8 parameters. (B) Statistical analysis of 8 parameters obtained from control and experimental groups designated as G1 to G8 (*n* = 6 rats). (C) Fluorescence images of Alexa-488-conjugated WGA and Alexa-594-stained GLUT4 of skeletal muscle sections from type 2 diabetic model rats after resting, walking, jogging, and fast running. (D) *t*-SNE for G1 to G8. The 4 ellipses indicate the confidence ellipse (95% confidence interval). (E) Flow block diagram of the closed-loop feedback mode for diabetes management.

We then analyzed the dynamic variation of *C*_glu_ in terms of 8 parameters (Fig. 6A), namely, the decrease rate of *C*_glu_ right after insulin injection but before exercise (*k*_1_), the decrease rate of *C*_glu_ during exercise (*k*_2_), the decrease rate of *C*_glu_ from the end of exercise to the minimum of curve (*k*_3_), the increase rate of *C*_glu_ from the minimum of curve to the glucose value after 180 min rest (*k*_4_), the maximum absolute variation of *C*_glu_ (Δ*C*_glu_), the integral variation of *C*_glu_ (AUC), the duration time of *C*_glu_ in the normal concentration range (*t*_1_), and the duration time of *C*_glu_ in the hypoglycemic concentration range (*t*_2_). Statistical analysis of 8 parameters for G1 to G8 is shown in Fig. 6B. *k*_1_ is related to the dose of injected insulin, so *k*_1_ in G5 to G8 is ~1.3-fold larger than that in G1 to G4. *k*_4_ is not that different as it is mainly determined by the individual differences among rats after insulin metabolism and exercise. *k*_2_, *k*_3_, Δ*C*_glu_, AUC, and *t*_1_ are closely associated with the insulin dose and exercise mode. At the same insulin dose, these paraments increase with the exercise intensity. For example, in G1 to G4, AUC increases from ~28.18 ± 2.08 mM h (G1) to ~29.84 ± 1.60 mM h (G2), ~33.48 ± 2.61 mM h (G3), and ~38.39 ± 1.90 mM h (G4), and *t*_1_ increases from ~0 min (G1) to ~14.76 ± 11.91 min (G2), ~64.68 ± 2.49 min (G3), and ~79.10 ± 5.36 min (G4). Similar trends are also found in G5 to G8. It means that the exercise can facilitate the decrease of *C*_glu_ and maintain *C*_glu_ in the normal glucose concentration range for a longer time. In the same exercise modes (G1 and G5; G2 and G6; G3 and G7; G4 and G8), the decrease of *C*_glu_ becomes sharp when increasing the insulin dose from 1 to 2 U kg^−1^ (Fig. 6B). In the case of jogging, AUC increases from ~33.48 ± 2.61 mM h (G3) to ~41.80 ± 2.93 mM h (G7) and *t*_1_ increases from ~64.68 ± 2.49 min (G3) to ~83.00 ± 4.93 min (G7). Moreover, the therapeutic effect of 1 U kg^−1^ insulin administration plus jogging is comparable to that of 2 U kg^−1^ insulin dose (G3 versus G5; AUC and *t*_1_ of G5 are ~34.83 ± 2.68 mM h and ~51.48 ± 5.52 min). However, overexercise likely causes the decline of *C*_glu_ into the hypoglycemic range, and the duration time of *C*_glu_ in this range is described by the parameter *t*_2_. *t*_2_ is negligible in G1 to G7 but remarkable in G8 (~23.04 ± 2.61 min). G8 can be considered as overexercise and is thus not recommended. The reason behind lies in the fact that the expression amount of GLUT4 on the skeletal muscle of rats increases with the exercise intensity. As seen in Fig. 6C and Fig. S27, the fluorescence intensity ratio of Alexa-594-stained GLUT4 to Alexa-488-conjugated wheat germ agglutinin (WGA)-stained cell membrane (GLUT4/WGA) on the muscle sections of diabetic model rats after jogging and fast running is 0.622 ± 0.072 and 1.057 ± 0.110, respectively. These 2 values are much larger than that of rats after walking (~0.282 ± 0.104) and resting (~0.185 ± 0.132), indicating that the skeletal muscle has indeed ingested and consumed more glucose.

According to the 8 parameters, the samples obtained from G1 to G8 were used as prototype and imported into an analytical database, which were then treated using the *t*-distributed stochastic neighbor embedding (*t*-SNE). As shown in Fig. 6D, the samples obtained from G1 to G8 can be segregated into 4 distinct cluster regions and every region represents different effect on the blood glucose control, namely, the gray region (upper left, G1 and G2) representing the ineffective treatment mode, the blue region (middle bottom, G3, G5, and G6) denoting the effective treatment mode, the pink region (lower right, G4 and G7) implying the treatment mode with potential risk of side effect, and the yellow region (middle right, G8) signifying the treatment mode with serious side effect. Based on this database, any blind sample can be identified and classified to analyze its rationality and output advice to adjust the treatment mode (Fig. 6E). For example, we selected a random mode (selected from G1 to G8) for a diabetic model rat. The obtained *C*_glu_ curve was input into the analytical procedure (sample 1 in Fig. S28). The analytical result shows that the Euclidean distance between sample 1 and samples in G8, namely, 1.35 ± 0.54, is the smallest, indicating the best similarity and the severe hypoglycemia during exercise (sample 1 in Fig. 6D) [48]. This conclusion matches well with the real treatment mode (2 U kg^−1^ insulin injection plus fast running for 30 min). In this case, the treatment mode was adjusted to avoid the side effect by decreasing the running speed to 5 m min^−1^ while maintaining the dose of insulin. The result was shown as sample 2 in Fig. 6D. The Euclidean distance between sample 2 and samples in G5 or G6 is 1.61 ± 0.34 or 3.50 ± 2.28, respectively. This treatment mode can avoid hypoglycemia and thus gain a good efficacy on the blood glucose control.

## Discussion

We report a closed-loop feedback strategy based on a MEP sensor for continuous detection of glucose in the ISF and an analytical procedure for data treatment to output the potency of exercise and give advice on exercise management. The MEP is based on the second-generation electrochemical glucose principle. Compared to previous MEP based on the first-generation electrochemical glucose principle [8], our sensor in principle has high selectivity, good anti-interference ability, and low operating potential. The MEP sensor is integrated with a battery-powered custom potentiostat to fabricate a microdevice, which can work stably on a freely moving rat and transmit the data wirelessly. The glucose signals at different insulin doses and exercise intensities can be real-timely recorded and analyzed using *t*-SNE and classified into different distinct cluster regions to evaluate the potency of exercise and insulin dose. This closed-loop feedback strategy can realize in vivo real-time detection of glucose level, wireless signal communication, analysis of the rationality of treatment mode, and advice for diabetic patients. We believe that it will provide plenty of opportunities to improve the personal diabetes management and the life quality of diabetic patients.

However, there are still a lot of challenges to scale up this strategy from the animal model to human. First, the epidermal and dermal thicknesses of human (~150 μm and ~2 mm) are much thicker than those of rat. The length and tip width of microneedles in the MEP need to be increased and reduced to ensure that the microneedles can be implanted into the dermis layer. Second, a soft and stretchable microneedle substrate should be employed to enhance user comfort for long-term skin adhesion. The diameter and number of microneedles need to be optimized to reduce the pain associated induced by the implantation of MEP. Third, our present preparation method is only appropriate for small batch production. Advanced technologies, such as 3D printing, vacuum sputtering, electroplating, ultrasonic spraying, and microfabrication, can be employed to scale up the manufacturing of the MEP for mass production, some of which have been indeed used in a previous work [8]. Moreover, the complex clinical trial needs to be performed to further investigate the biosafety and stability on human. At last, because of the physiological differences (such as height, weight, age, and metabolic level) among individuals, our closed-loop feedback model needs to be corrected or rebuilt.

## Materials and Methods

### Materials

All chemicals and reagents were of analytical grade or higher and were used as received without further purification (details are given in Note S1).

### Animals

Male Sprague–Dawley (SD) rats (≈200 to 300 g in weight, 6 to 7 weeks old) were purchased from the Animal Experiments Center of Zhejiang University (ZJU). All experiments were performed in accordance with the Guidelines for the Care and Use of Laboratory Animals of ZJU and approved by the Animal Advisory Committee of ZJU (code: ZJU 20220264). Rats were housed at a constant temperature (≈25 °C) and relative humidity (≈60%) under a 12-h light/dark schedule with food and water ad libitum. The rats were randomly assigned to the experimental groups. During the data collection, the experimenters were not blinded to the experimental groups or conditions.

### Preparation of MEP

Briefly, PS solution (0.1 g ml^−1^ in dichloromethane) was added to the poly(dimethyl siloxane) (PDMS) mold to fill the pyramidal holes under vacuum, followed by drying at room temperature. PS microneedle array was peeled off from the mold and tailored into small pieces. Then, the conductive layer was deposited on PS microneedle array by electroless deposition. The obtained MEA was used as the CE directly. To prepare the MEA for glucose detection (gMEA) and RE, corresponding solutions were coated on MEA. Finally, CE, RE, and gMEA were integrated onto a flexible polyimide (PI) circuit board via silver conducting resin to prepare the MEP. Further details on the preparation and characterization of MEP are provided in Note S2.

### Electrochemical measurements

Electrochemical measurements were conducted on CHI660D workstation or with a custom wireless electrochemical device. The room temperature is strictly at 25 °C for in vitro electrochemical measurements.

### Histology

The biocompatibility of MEP was studied by histology. Rats were anesthetized with 2% isoflurane. Then, the MEP was implanted into the skin of back and fixed with Tegaderm film (3M, USA). After implanting MEP, rats were anesthetized and decapitated to isolate the skin of back, heart, liver, spleen, lung, and kidney. The isolated skin and organs were fixed by 4% formalin for 48 h and then embedded in paraffin. Sections with a thickness of 4 μm were cut using paraffin slicing machine (Leica RM2235). Sections were treated with H&E staining solution, subsequently mounted with the neutral balsam, and observed on an optical microscope (Olympus CX21).

### Type 2 diabetic model rats

The type 2 diabetic model rat was established by high-fat diet and streptozotocin administration. Briefly, rats were first fed with high-fat diet for 2 weeks. Then, 0.1 mM sodium citrate buffer containing 10 mg ml^−1^ streptozotocin (pH 4.5) was injected into the abdominal cavity of rats (40 mg kg^−1^, once a day) for 2 d. To confirm the establishment of diabetic model, the blood glucose value of rats was measured with a commercial glucometer (Sinocare, Changsha) by collecting blood from the tail vein.

### In vivo electrochemical detection of glucose with MEP

The wireless electrochemical device consists of a MEP, a custom miniaturized potentiostat, and a lithium battery. A low-power microcontroller was used to program the required excitation potentials and retrieve readout signals. For chronoamperometry measurements, voltage was applied to the electrode using a digital-to-analog converter. The current was first converted to a voltage signal through a transimpedance amplifier. Then, the data of voltage signal were collected and temporarily stored into a Bluetooth packet using a successive approximation analog-to-digital converter with a sampling rate of 2 Hz. Finally, the data were transmitted to the user interface on a smart phone through a Bluetooth transceiver with a transmitting rate of 1 Hz. The Advanced Encryption Standard 128 bit (AES-128) encryption algorithm was used to ensure the security and privacy of the data transmitted from the device to the smartphone. The MEP is disposable, and its usage time is strictly controlled within 24 h.

### Exercise management

Rats were habituated to run on the KW-PT treadmill (NJKEWBIO, Nanjing), and a stainless-steel grid at the end of the treadmill could supply an electrical stimulus to force the rats running. The magnitude and duration of the current were 0.8 mA and 5 s, respectively. Only rats that could run at an appropriate speed (5 to 25 m min^−1^) for 30 min were selected to establish the type 2 diabetic model rats. Moreover, only type 2 diabetic model rats with the blood glucose level ranging from 20 to 25 mM were used in the following experiment. The selected rat was anesthetized with 2% isoflurane, then MEP was implanted into the skin of back, and the custom wireless electrochemical device was fixed with Tegaderm film. After waiting for 30 min to obtain a stable current signal, insulin solution (1 or 2 U kg^−1^) was injected into the abdominal cavity, and 1 h later, the rat was forced to run at different speeds (5, 15, or 25 m min^−1^) on the treadmill for 30 min. The MEP needed to be calibrated once after being implanted into the skin. Briefly, amperometric currents were recorded for 60 s every 10 min and the blood glucose concentrations were measured every 15 min for 1 h to establish the correlation between current and blood glucose concentration.

### Immunohistochemistry of GLUT4

To analyze GLUT4 translocation, type 2 diabetic model rats after rest or exercise at a speed of 5, 15, and 25 m min^−1^ for 30 min on the treadmill (designated as resting, walking, jogging, and fast running group, respectively) were immediately anesthetized and decapitated to isolate the gastrocnemius muscle. The muscle was sequentially fixed by 4% formalin for 48 h and embedded in paraffin. Sections with a thickness of 4 μm were cut using paraffin slicing machine. They were first incubated with rabbit polyclonal antibody against GLUT4 (Thermo Fisher Scientific, PA5-23052; antibody was diluted in 1× PBS to 10 μg ml^−1^) overnight at 4 °C, followed by 10 min of washing for 3 times, and the second incubation for 1 h with goat anti-rabbit F(ab′)_2_ fragment-specific antibody conjugated to Alexa-594 (Jackson ImmunoResearch, 111-585-047; antibody was diluted in 1× PBS to 3.75 μg ml^−1^). Then, sections were washed 10 min for 3 times in 1× PBS and incubated for 15 min with WGA conjugated to Alexa-488 diluted to 5 μg ml^−1^ in 1× PBS [49]. To quantify the expression of GLUT4 on the cell surface, fluorescence images were captured on Olympus microscope (BX63, Olympus). The relative amount of GLUT4 on the cell membrane for each group was calculated as the fluorescence intensity ratio of GLUT4 to WGA staining (GLUT4/WGA) in a line drawn perpendicular through the cell membrane. The fluorescence intensity ratio of GLUT4/WGA increased with the increase of running speed of rats. Walking barely affects GLUT4 translocation in comparison with the resting group. Jogging and fast running will significantly promote GLUT4 translocation, thus increasing the glucose uptake by muscle.

### Statistical analyses

Statistical analyses were performed using Microsoft Excel 2019 (Microsoft Corporation, USA). Data from failed experimental groups were excluded from the statistical analysis. All data were expressed as mean ± standard deviation, unless noted otherwise. No statistical methods were used to predetermine the sample size, which was annotated in the context or figure captions. For 2-group comparisons, statistical significance was determined using a 2-tailed unpaired Student’s *t*-test. Statistical significance was set at *P* < 0.05. Statistically significant results are indicated in the figures using *P < 0.05, **P < 0.01, ***P < 0.001, ****P < 0.0001, and no significance (n.s., *P* > 0.05).

## Data Availability

The authors confirm that the data supporting the findings of this study are available within the article and/or its Supplementary Materials. The data can be obtained from the corresponding author upon reasonable request.

## Supplementary Materials

Supplementary 1 Notes S1 to S3 Figs. S1 to S28 Movies S1 and S2
