# Supplementary material for: A Wearable Integrated Microneedle Electrode Patch for Exercise Management in Diabetes
Source: Research (Wash D C). 2024 Oct 21;7:0508. doi: 10.34133/research.0508 (PMC11491670; doi:10.34133/research.0508)
Supplement: Supplementary 1 — Notes S1 to S3 Figs. S1 to S28 Movies S1 and S2 [file research.0508.f1.zip › SI-RESEARCH-MN-cleancopy.docx]

**Supplementary Information**

**Title**

A Wearable Integrated Microneedle Electrode Patch for Exercise Management in Diabetes

**Authors**

Boyu Zhu^1^^†^, Lihang Zhu^2†^, Xinru Li^1^, Ziyi Zhao^1^, Jiayi Cao^1^, Min Qi^1^, Zhigang Gao^3^*, Lin Zhou^1^*, Bin Su^1,3^*

**Affiliations**

^1^Institute of Analytical Chemistry, Department of Chemistry, Zhejiang University, Hangzhou, 310058, China

^2^Department of Clinical Engineering, Second Affiliated Hospital, College of Medicine, Zhejiang University, Hangzhou 310009, China

^3^General Surgery Department, Children’s Hospital, Zhejiang University School of

*Address correspondence to: Zhigang Gao, ebwk@zju.edu.cn; Lin Zhou, zhoulinchem@zju.edu.cn; Bin Su, subin@zju.edu.cn.

^†^These authors contributed equally to this work.

**Note S1: Chemicals and materials**

**Chemicals and reagents**

All chemicals and reagents were of analytical grade or higher and used as received without further purification. All aqueous solutions were prepared with ultrapure water (18.2 MΩ cm, Milli-Q, Millipore). Polystyrene was brought from NantongFeiyu Biological Technology. Tin (II) chloride (SnCl_2_, 97.5%) was bought from J&K Scientific. Commercial gold electroless plating solution containing 0.25 M gold (I) trisodium disulphite [Na_3_Au(SO_3_)_2_] was ordered from Changzhou Institute of Chemical Research. D-(+)-glucose (C_6_H_12_O_6_, ≥99.5%), 1-vinylimidazole (C_5_H_6_N_2_, 99%), 2,2’-azobis(2-methylpropionitrile) (AIBN, C_8_H_12_N_4_, 98%), 2,2’-bipyridine (C_10_H_8_N_2_, 99.0%), paraformaldehyde (PFA, 95%), poly(ethylene glycol) diglycidyl ether [PEGDGE, C_3_H_5_O_2_-(C_2_H_4_O)_n_-C_3_H_5_O], trifluoroacetic acid (CF_3_COOH, 99.5%), silver nitrate (AgNO_3_, 99.8%), trisodium citrate dihydrate (Na_3_C_6_H_5_O_7_⋅2H_2_O, 98%), citric acid monohydrate (C_6_H_8_O_7_⋅H_2_O, 99.5%), ascorbic acid (C_6_H_8_O_6_, 99%), uric acid (C_5_H_4_N_4_O_3_, 99%), lactic acid (C_3_H_6_O_3_, 85%), urea (CH_4_N_2_O, 98%), acetaminophen (C_8_H_9_NO_2_, 99%), polyvinyl butyral (PVB, C_8n_H_14n+2_O_2n_, 15.0−18.0 mPa⋅s), potassium ferricyanide (III) [K_3_[Fe(CN)_6_], 99.0%], agarose (C_12_H_18_O_9_, 99%) and Tris-hydrochloride buffer (1 M, pH = 8.5) were purchased from Aladdin. Silver/silver chloride (Ag/AgCl) ink was brought from Julon Technology. Glucose oxidase (GOx, A600243, 100 Units mg^−1^) was ordered from Sangon Biotech. Nafion (5 wt% in lower aliphatic alcohols and water, containing 15−20% water) and ammonium hydroxide solution (NH_3_⋅H_2_O, 25 wt%) were bought from Sigma-Aldrich. Methanol (CH_4_O, 99.5%), ethanol (C2H6O, ≥99.7%), formaldehyde solution (HCHO, 37−40%), dichloromethane (CH_2_Cl_2_, ≥99.5%), ethylene glycol (C_2_H_6_O_2_, 99.5%), acetone (C_3_H_6_O, 99.5%), diethyl ether (C_4_H_10_O, 99.5%), sodium sulfite (Na_2_SO_3_, 96%), sodium chloride (NaCl, 99.5%), potassium chloride (KCl, 99.5%), ammonium chloride (NH_4_Cl, 99.5%), sodium phosphate dibasic dodecahydrate (Na_2_HPO_4_⋅12H_2_O), potassium dihydrogen phosphate (KH_2_PO_4_, 99.5%), hydrochloric acid (HCl, 36.0−38.0%), sodium hydroxide (NaOH, ≥96%) and sulfuric acid (H_2_SO_4_, 95.0−98.0%) were purchased from Sinopharm. Ammonium hexachloroosmate (IV) [(NH_4_)_2_OsCl_6_, 99%], sodium dithionite (Na_2_S_2_O_4_, 90%) and streptozotocin (C_8_H_15_N_3_O_7_, 98%) were ordered from Macklin. Insulin from bovine pancreas (27 Units mg^−1^) was purchased from Solarbio Life Sciences. Poly(dimethyl siloxane) (PDMS) was bought from Dow Corning. Hematoxylin (BL702A) and eosin (BL703A) were received from Biosharp. The PDMS mold for fabrication of microneedle was brought from Microchip Pharmaceutical Technology. 1× Phosphate buffered saline (PBS) and rabbit polyclonal antibody against glucose transporter type 4 (GLUT4) were ordered from Thermo Fisher Scientific. Goat anti-rabbit F(ab’)2 fragment-specific antibody conjugated to Alexa-594 was brought from Jackson ImmunoResearch. Wheat germ agglutinin (WGA) conjugated to Alexa-488 was ordered from AAT Bioquest. 0.1 M phosphate buffer (PB) was prepared by adding 80 mmol of Na_2_HPO_4_⋅12H_2_O and 20 mmol of KH_2_PO_4_ to 1.0 L of ultrapure water. The pH of PB was adjusted to 7.2−7.4 by the addition of concentrated HCl and NaOH.

**Synthesis of osmium-derivatized poly(1-vlnylimidazole) (PVI-Os)**

Briefly, poly(1-vinylimidazole) (PVI) was first prepared by heating the mixture containing 0.5 g of AIBN and 6 mL of 1-vinylimidazole at 70 ^o^C for 2 h under N_2_. Osmium (II) bis(2,2-bipyridine) dichloride [Os(bpy)_2_Cl_2_] was prepared by heating 1.0 g of (NH_4_)_2_OsCl_6_ and 0.72 g of 2,2’-bipyridine in 50 mL of ethylene glycol at 205 ^o^C for 45 min under N_2_. Then, PVI-Os was synthesized by heating 115 mg of PVI and 210 mg of Os(bpy)_2_Cl_2_ in 150 mL of ethanol at 80 ^o^C for 3 days under N_2_.

**Skin-mimicking agarose gel**

To prepare the skin-mimicking agarose gel, 0.4 g of agarose powder was mixed with 20 mL of PBS containing glucose, which was kept at 120 ^o^C until the complete dissolution of agarose. Then the solution was poured into culture dish and cooled to room temperature to form gel.

**Note S2: Preparation and characterization of microneedle electrode patch (MEP)**

**Preparation of microneedle electrode patch (MEP)**

The preparation of MEP involves five steps (Fig. S2).

Step 1: Preparation of polystyrene (PS) microneedle array. PS microneedle array was prepared using a PDMS mold, which consisted of pyramidal holes with a depth of ~1000 μm, a width of ~500 μm at the orifice and a center-to-center distance of ~800 μm (Fig. S2A). The PS solution (0.1 g mL^−1^ in dichloromethane) was added to the mold to fill the holes under vacuum. After being dried at room temperature for 12 h, the PS microneedle array was peeled off from the PDMS mold and tailored into small pieces. The number of microneedles is 4 × 10 in each small piece.

Step 2: Preparation of microneedle electrode array (MEA). The thin gold layer was deposited on PS microneedle array by electroless deposition. As shown in Fig. S2B, the PS microneedle array was first immersed in methanol for 2 h, which was subsequently sensitized with Sn^2+^ in a methanol solution containing 0.026 M of SnCl_2_ and 0.07 M of trifluoroacetic acid for 45 min. After rinsing with methanol, the PS microneedle array was immersed in an aqueous solution containing 0.029 M of [Ag(NH_3_)_2_]NO_3_ for 10 min, which was freshly prepared by dropwise addition of concentrated NH_3_⋅H_2_O into the aqueous solution containing 0.029 M of AgNO_3_. Finally, the PS microneedle array was immersed in commercial gold electroless plating solution containing 0.127 M of Na_2_SO_3_, 0.0148 M of Na_3_Au(SO_3_)_2_ and 0.25 M HCHO at 4 ^o^C for 24 h. The microneedle array was rinsed with deionized water and dried under room temperature. Thus obtained microneedle electrode array (designed as MEA) was used as the counter electrode (CE) directly.

Step 3: Preparation of microneedle electrode array for glucose detection (gMEA). 20 μL of solution containing 0.3 mg mL^−1^ of PVI-Os, 1.7 mg mL^−1^ of PEGDGE and 3.3 mg mL^−1^ of GOx was coated on MEA. After drying at 4 ^o^C for 12 h, 20 μL of 0.5% Nafion was further coated (Fig. S2C). Thus obtained MEA coated with Nafion/GOx/PVI-Os/PEGDGE layer was designed as gMEA in the context.

Step 4: Preparation of reference electrode (RE). MEA was firstly immersed in Ag/AgCl ink. After drying at 60 ^o^C for 30 min, it was dip coated with 20 μL of methanol solution containing 79.1 mg mL^−1^ of PVB and 50 mg mL^−1^ of NaCl and dried at room temperature. Thus obtained MEA coated with PVB/NaCl/AgCl/Ag was used as the reference electrode (RE).

Step 5: The assembly of MEP. The MEP was prepared by integrating CE, RE and gMEA onto a flexible polyimide (PI) circuit board via silver conducting resin.

To evaluate the water swelling of different microneedle arrays (CE, RE and gMEA), we simulated the in vivo application environment and immersed the arrays in PBS for 4 h. The weight of arrays was measured for different durations (0, 1, 2, 3, and 4 h).

The mechanical properties of microneedles were measured using a TA-XT plus texture analyzer. Microneedle electrode array was stuck onto a rigid platform and pressed by a sensor probe (*d* = 5 mm) in the vertical direction with a speed of 0.01 mm s^−1^. The displacement force was recorded when the sensor probe touched the microneedle tips.

**Note S3: In vitro electrochemical detection of glucose with MEP**

Glucose can react with Os^3+^ under the catalysis by GOx (Equations S1−S3):

| $\text{glucose + GOx(FAD)}\to\text{gluconolactone + GOx(FADH}_{2}\text{)}$ | (S1) |
| --- | --- |
| $\text{GOx(FADH}_{2}\text{) + 2PVI-Os}^{3+}\to\text{GOx(FAD) + 2PVI-Os}^{2+}$ | (S2) |
| $\text{PVI-Os}^{2+}\to PVI\text{-Os}^{3+}\text{ + e}^{-}$ | (S3) |

The oxidation current recorded with MEP increases with increasing the concentration of glucose, matching with the Michaelis-Menten kinetics model (Fig. S12). The apparent Michaelis-Menten constant (*K*_m_) representing the enzyme-substrate kinetics can be calculated by the Lineweaver-Burk equation (Equation S4).

| $\frac{1}{I}=\frac{K_{m}}{I_{\max}}\times\frac{1}{C}+\frac{1}{I_{\max}}$ | (S4) |
| --- | --- |

where *I* and *I*_max_ are the steady-state and the maximum current obtained with MEP. *C* is the concentration of glucose.


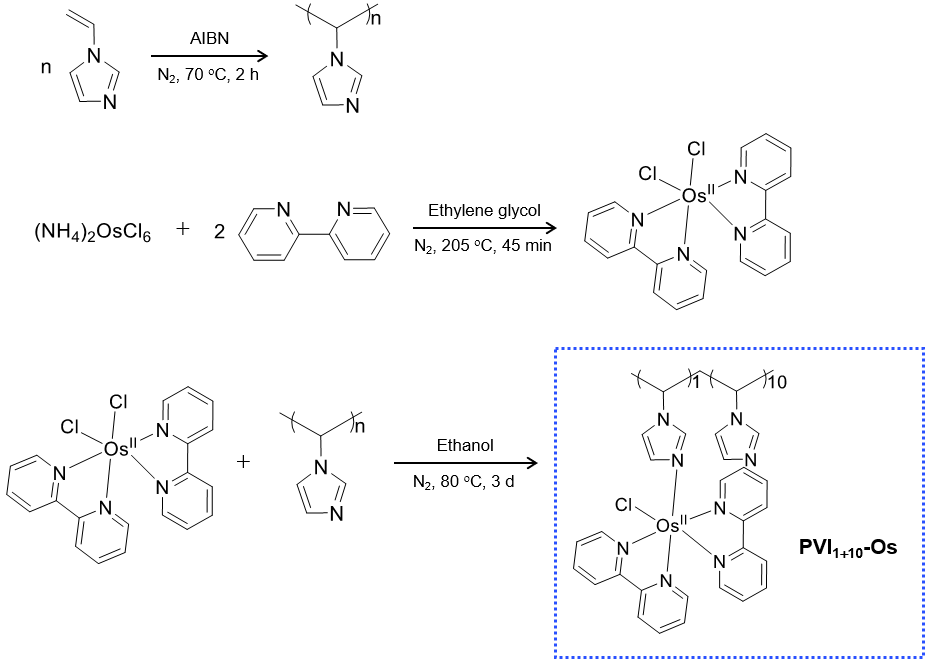


**Fig. S1.** Schematic illustration of preparation of PVI-Os.


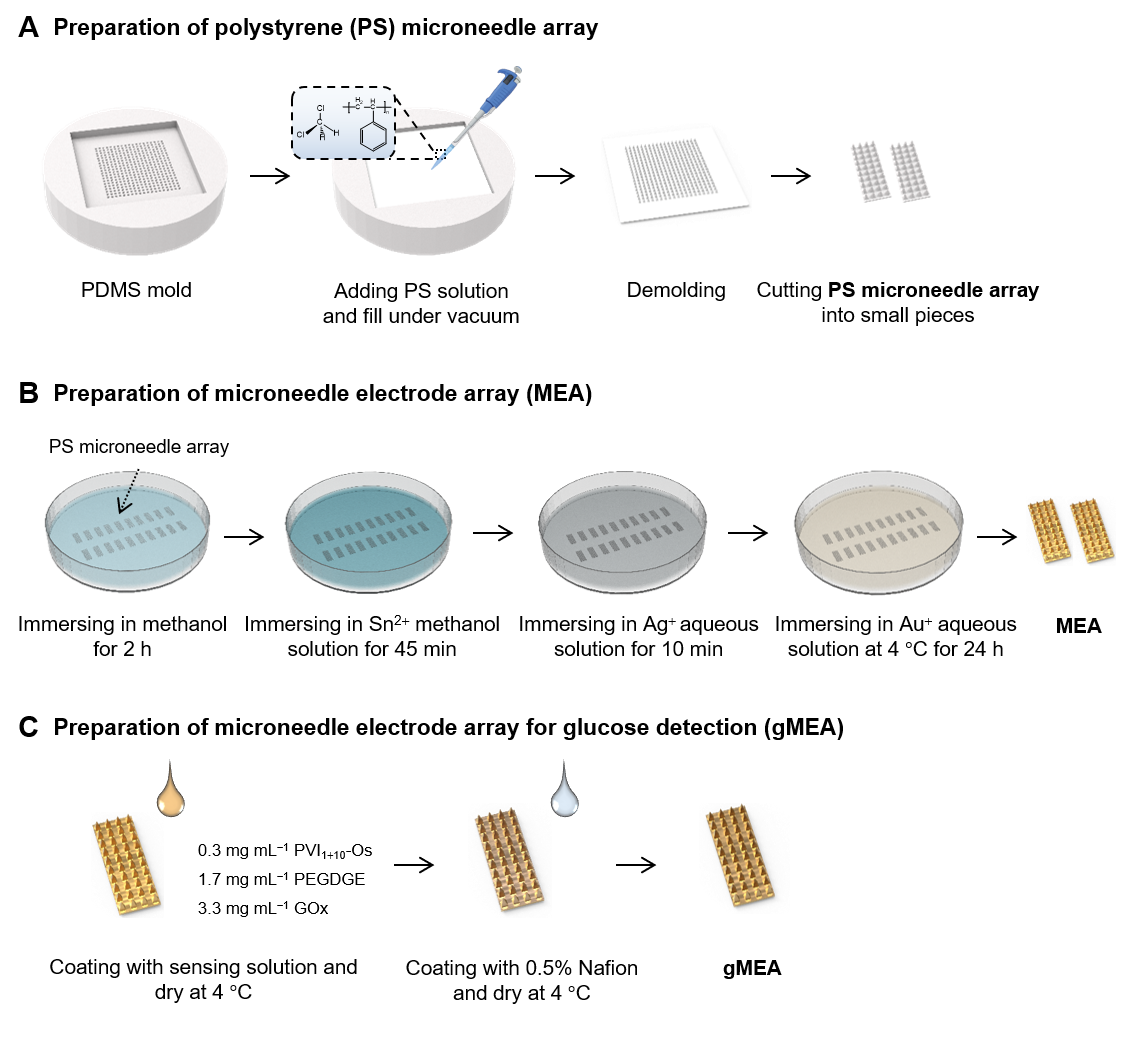


**Fig. S2.** Schematic illustration of preparation of microneedle array. Schematic illustration of preparation of polystyrene microneedle array (A), microneedle electrode array (MEA) (B) and microneedle electrode array for glucose detection (gMEA) (C).


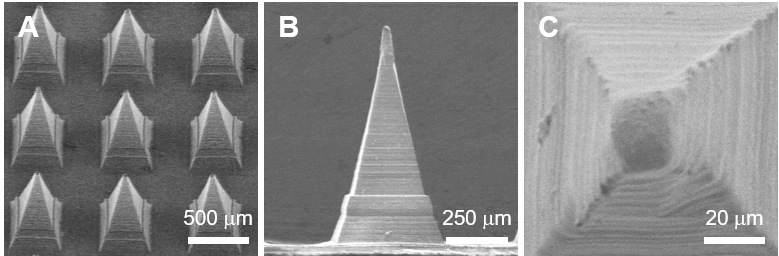


**Fig. S3.** Scanning electron microscopy (SEM) image of PS microneedles. (A) Top-view SEM image of PS microneedle array. (B, C) Side-view SEM image (B) and high-magnification SEM image of the tip (C) of a single PS microneedle.


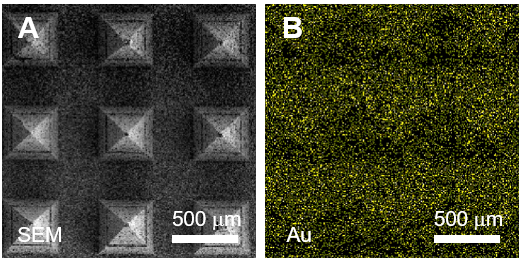


**Fig. S4.** Energy dispersive X-ray spectroscopy (EDX) analysis of MEA. SEM image of MEA (A) and energy dispersive X-ray spectroscopy (EDX) analysis of Au (B) element on MEA.


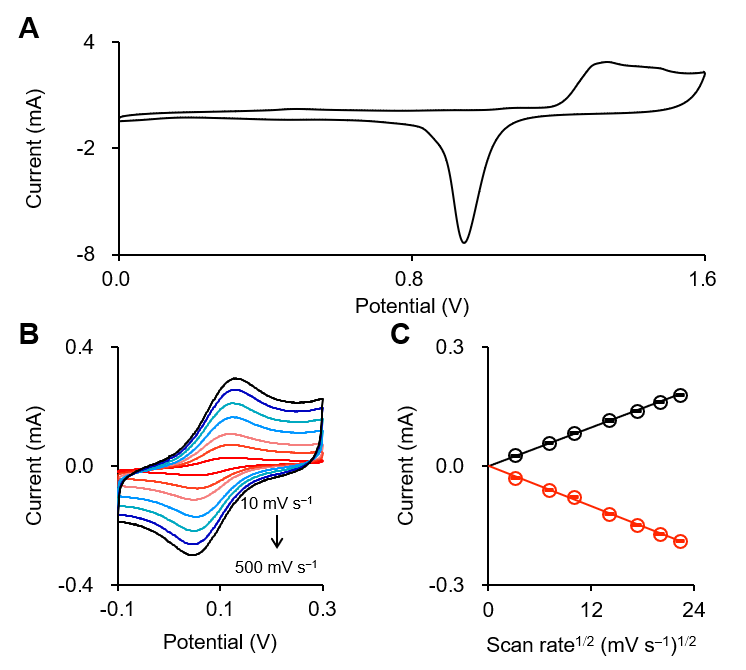


**Fig. S5.** Characterization of MEA. (A) Cyclic voltammograms (CVs) obtained with MEA in 1.0 M H_2_SO_4_ solution. The scan rate was 100 mV s^−1^. (B) CVs obtained with MEA in 0.1 M PB containing 1 mM K_3_[Fe(CN_6_)] at different scan rates from 10 to 500 mV s^−1^ (10, 50, 100, 200, 300, 400, 500 mV s^−1^). (C) The dependence of oxidation (black) and reduction peak currents (red) on the square root of scan rates (*n* = 3 times). Data are expressed as mean ± the standard deviation.


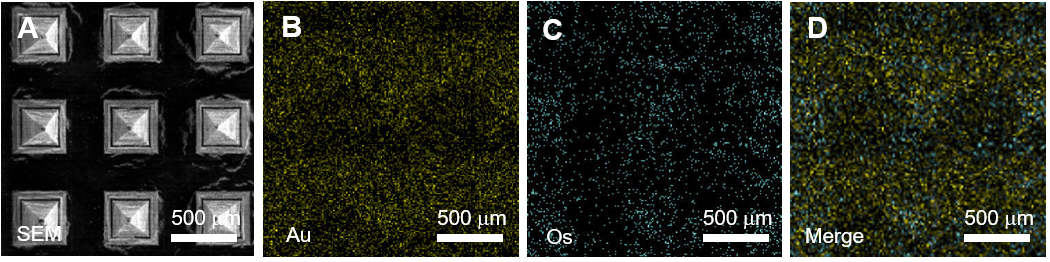


**Fig. S6.** EDX analysis of gMEA. SEM image of gMEA (A) and EDX mapping of Au and Os elements on gMEA (B−D).


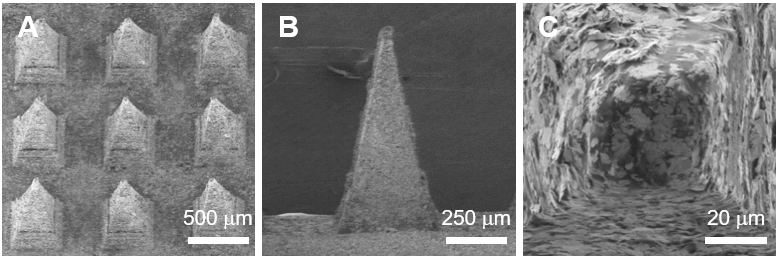


**Fig. S7.** SEM images of RE. (A) Top-view SEM image of RE. (B, C) Side-view SEM image (B) and high-magnification SEM image of the tip (C) of a single microneedle in RE.


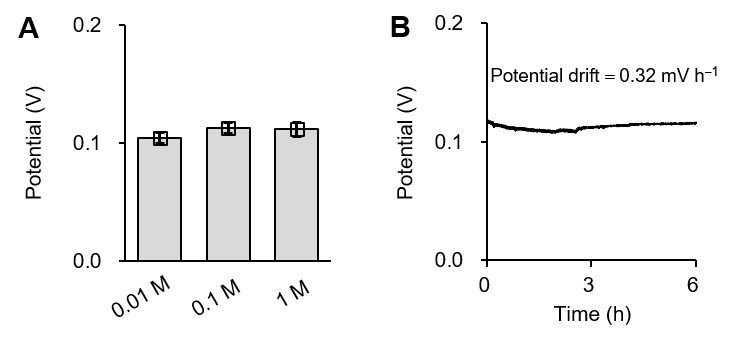


**Fig. S8.** The stability of RE. (A) The electrode potential (vs. Ag/AgCl/saturated KCl solution) obtained with RE in 0.01, 0.1 and 1 M NaCl solution (*n* = 3 times). Data are expressed as mean ± the standard deviation. (B) The electrode potential (vs. Ag/AgCl/saturated KCl solution) obtained with RE in saline solution over 6 h.


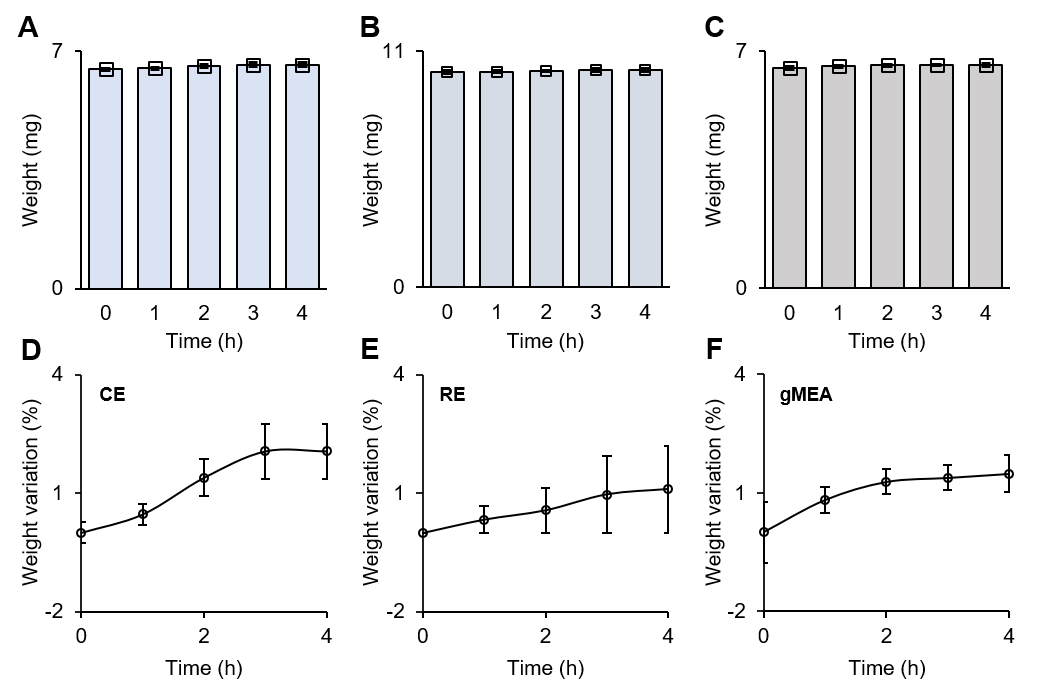


**Fig. S9.** The swelling behavior of microneedles. The weight of CE (A), RE (B) and gMEA (C) immersed in PBS for different durations (*n* = 3 electrodes) and the corresponding weight variations of CE (D), RE (E) and gMEA (F) immersed in PBS for different durations (*n* = 3 electrodes). Data are expressed as mean ± the standard deviation.


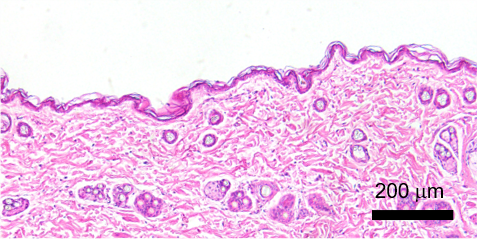


**Fig. S10.** Optical image of H&E stained skin section without MEP implantation.


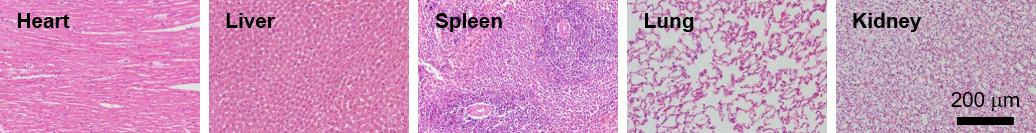


**Fig. S11.** Optical images of H&E stained heart, liver, spleen, lung and kidney sections from rats implanted with MEP for 7 days.


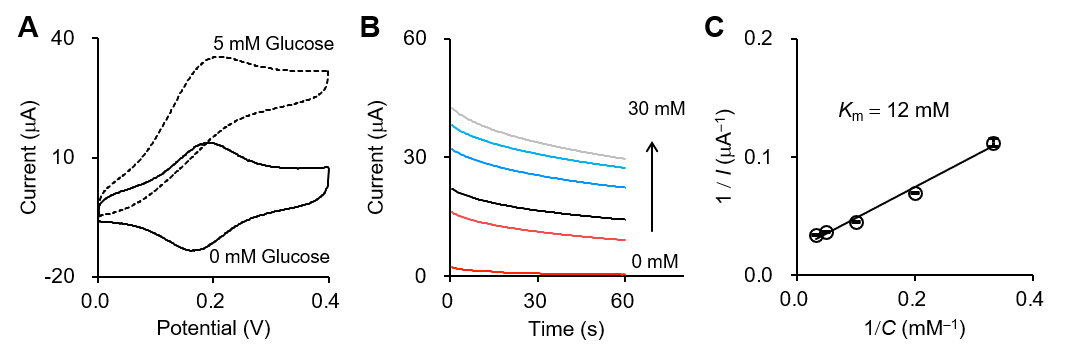


**Fig. S12.** Electrochemical detection of glucose with MEP in PBS solution. (A) CVs obtained with MEP in PBS solution containing 0 (solid curve) and 5 mM (dashed curve) glucose. The scan rate was 10 mV s^−1^. (B) Chronoamperometric curves recorded with MEP in PBS solution containing different concentrations of glucose: 0, 3, 5, 10, 20 and 30 mM from bottom to top. The electrode potential was biased at +0.2 V. (C) Lineweaver-Burk plot of 1/*I* vs 1/*C* (*n* = 3 times). *I* is the steady-state current obtained with MEP in PBS solution and *C* is the concentration of glucose.


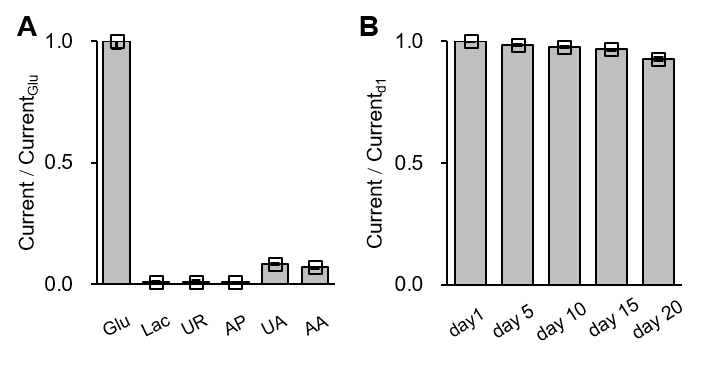


**Fig. S13.** Selectivity and stability of MEP. (A) Relative amperometric responses obtained with MEP in PBS solution containing 5 mM glucose (Glu), 5 mM lactic acid (Lac), 5 mM urea (UR), 200 μM acetaminophen (AP), 200 μM uric acid (UA), 100 μM ascorbic acid (AA) (*n* = 3 times). The electrode potential was biased at +0.2 V. Data are expressed as mean ± the standard deviation. (B) Relative amperometric responses obtained with MEP in PBS solution containing 5 mM glucose after being stored under nitrogen atmosphere at 4 ^o^C for ~3 weeks (*n* = 3 times). The electrode potential was biased at +0.2 V. Data are expressed as mean ± the standard deviation.


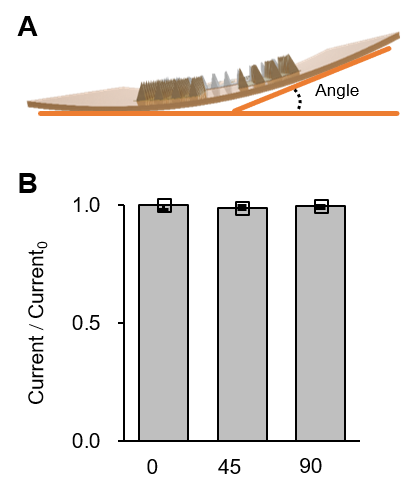


**Fig. S14.** Flexibility of MEP. (A) Schematic illustration of the bending test for MEP. (B) Relative amperometric responses obtained with MEP in PBS solution containing 5 mM glucose with bending at different angles (*n* = 3 times). The electrode potential was biased at +0.2 V. Data are expressed as mean ± the standard deviation.


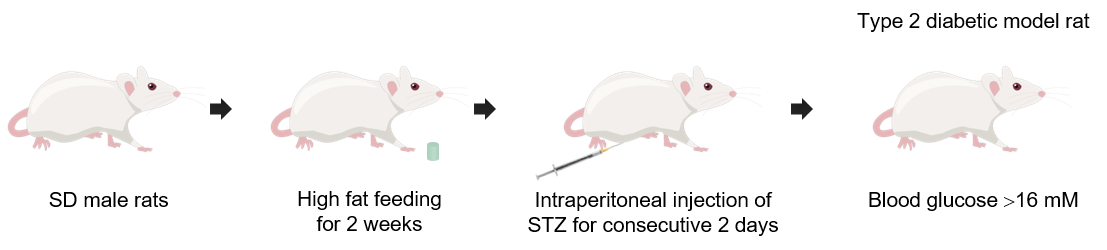


**Fig. S15.** The establishment of type 2 diabetic model rat.


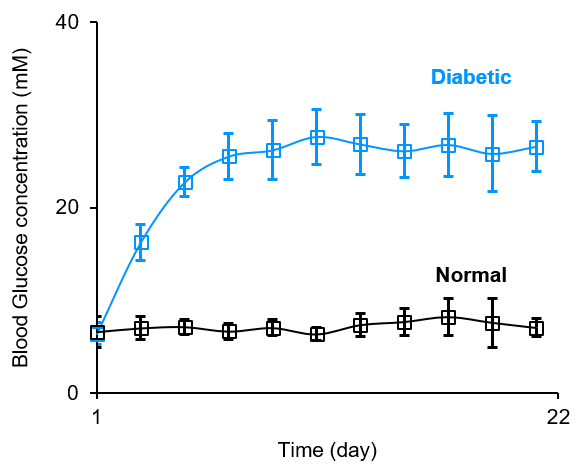


**Fig. S16.** Blood glucose concentration curves. Comparison of daily measured blood glucose concentrations of normal rats and type 2 diabetic model rats (*n* = 6 rats). Data are expressed as mean ± the standard deviation.


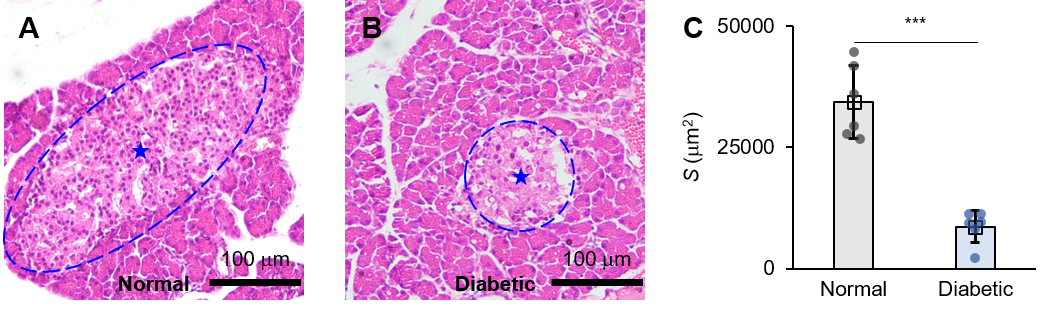


**Fig. S17.** Histology of pancreas. (A, B) Optical images of H&E stained tissue sections from normal (A) and type 2 diabetic model (B) rat pancreas. The blue dashed lines indicate the location of islet. (C) Statistical analysis of the area of islet (*S*) (*n* = 6 rats). Data are expressed as mean ± the standard deviation. Significance was determined by two-tailed unpaired Student’s *t*-test (****p* < 0.001).


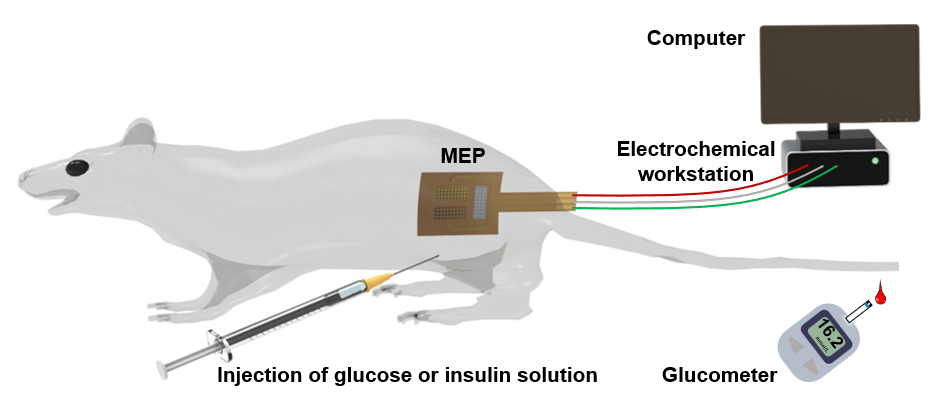


**Fig. S18.** Correlation between the current of MEP and blood glucose concentration. Concurrent measurements of the current response of implanted MEP and the glucose concentration in blood from tail vein by a commercial glucometer. The glucose concentrations in ISF and blood of rats were varied by injecting glucose or insulin solution to the abdominal cavity for normal rats or type 2 diabetic model rats, respectively.


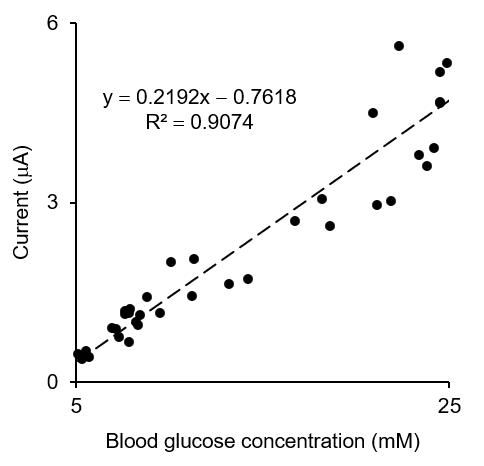


**Fig. S19.** The linear correlation between current signal of MEP and blood glucose concentration.

**Fig. S20.** Schematic of the circuit board. The electronic components include the STM32L432KBU6, the TPS73633, the AD8605ARTZ, the BLE, and the passive components.

**Fig. S21.** PCB layout of the circuit board.


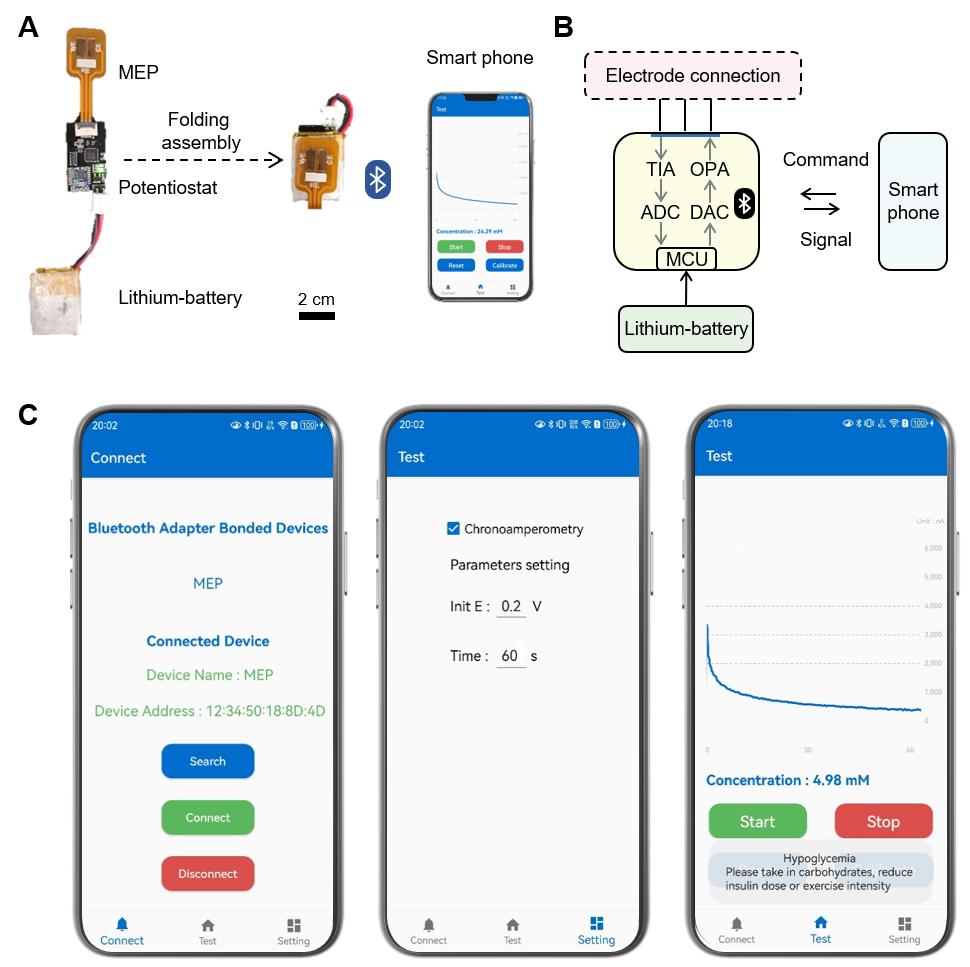


**Fig. S22.** Custom wireless electrochemical device. (A) Photograph of the wireless device. (B) Block diagram and workflow of the wireless electrochemical device. (C) The user interface of the smartphone app.


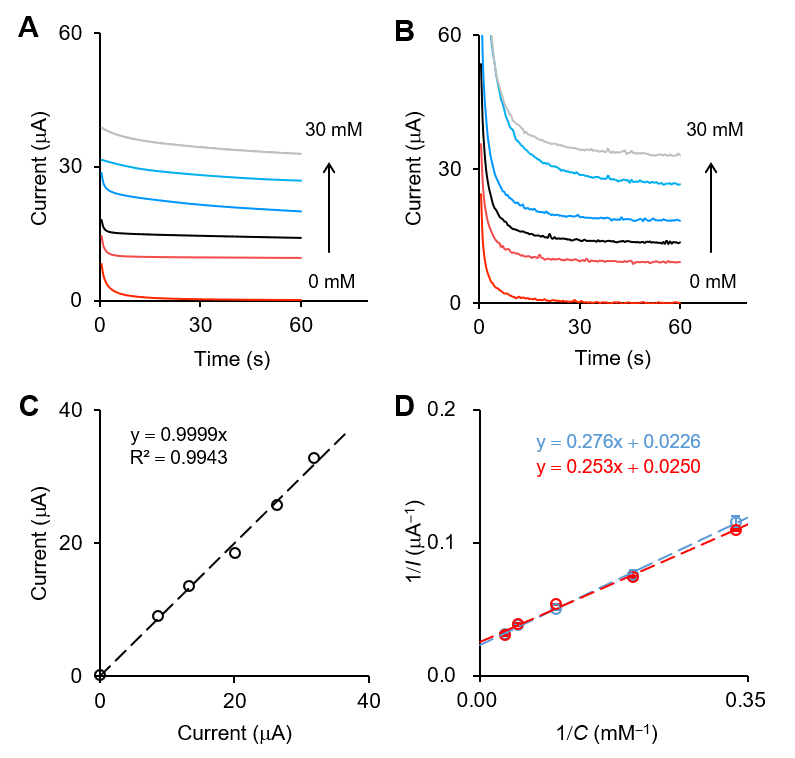


**Fig. S23.** Reliability of custom wireless electrochemical device. (A, B) Chronoamperometric curves recorded with MEP in PBS solution containing different concentrations of glucose (0, 3, 5, 10, 20, 30 mM glucose, from bottom to top) using CHI660D electrochemical workstation (A) and custom wireless electrochemical device (B). The electrode potential was biased at +0.2 V. (C) The linear correlation of current values measured in two cases. (D) The Lineweaver-Burk plots of 1/*I* vs 1/*C* obtained in two cases (blue: CHI660D electrochemical workstation; red: custom wireless electrochemical device) (*n* = 3 times). *I* is the steady-state current obtained with MEP in PBS solution and *C* is the concentration of glucose.


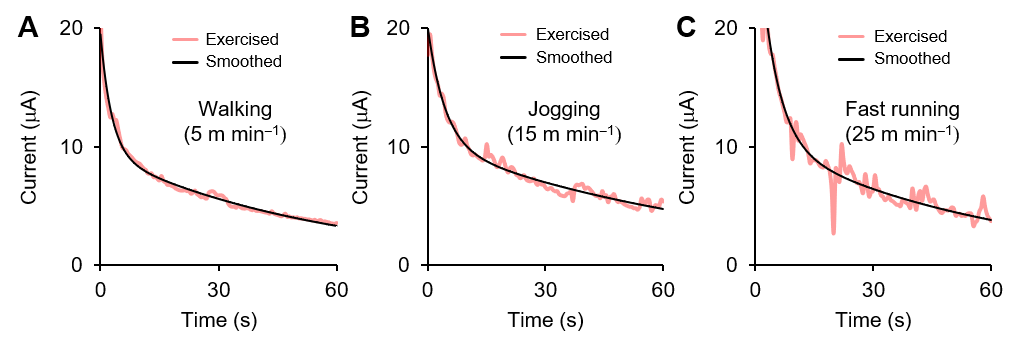


**Fig. S24.** Stability of custom wireless electrochemical device on running rats. Chronoamperometric curves recorded by the custom wireless electrochemical device for type 2 diabetic model rats running at different speeds (5, 15, and 25 m min^−1^) (A−C). Red curves represent the original chronoamperometric curves and black curves the smoothed ones. The electrode potential was biased at +0.2 V.


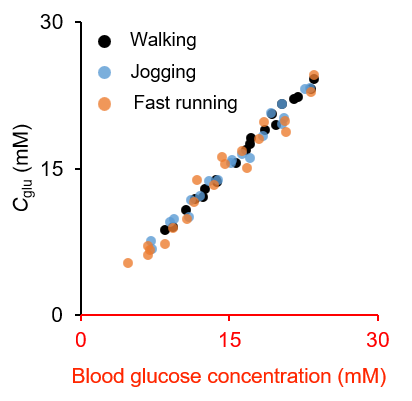


**Fig. S25.** Correlation between the blood glucose concentration of rats determined by FreeStyle Libre glucose sensor and the *C*_glu_ measured by the custom wireless electrochemical device on rats running at different speeds.


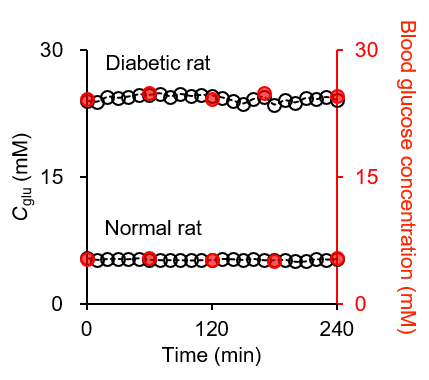


**Fig. S26.** Long-term stability of custom wireless electrochemical device on rats. Comparison of *C*_glu_ determined by MEP and custom wireless electrochemical device (black) for freely-moving normal and type 2 diabetic model rats and the blood glucose concentration measured by commercial glucometer (red) at different time over 240 min. For the measurement by custom wireless electrochemical device and commercial glucometer, the test was conducted every 10 and 60 min, respectively.


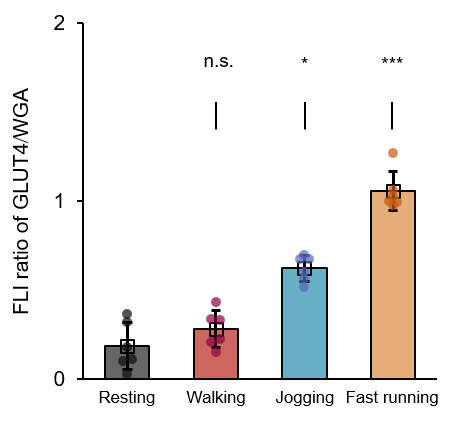


**Fig. S27.** Statistical analysis the fluorescence intensity. The fluorescence intensity (FLI) ratio of Alexa-594 stained GLUT4 to Alexa-488-conjugated WGA stained cell membrane (GLUT4/WGA) on the gastrocnemius muscle sections isolated from type 2 diabetic model rats of resting group and different experimental groups after exercise for 30 min (*n* = 6 sections).


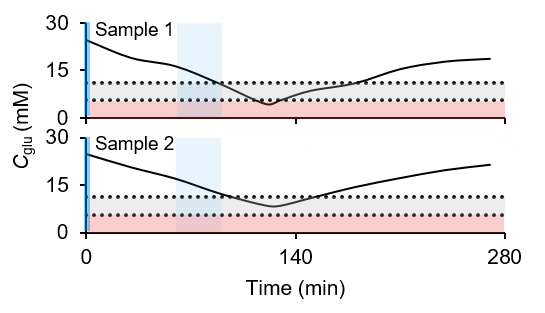


**Fig. S28.** The variations of *C*_glu_ of sample 1 and sample 2.
